# Whole genome integrity and enhanced developmental potential in ram freeze-dried spermatozoa at mild sub-zero temperature

**DOI:** 10.1038/s41598-020-76061-x

**Published:** 2020-11-02

**Authors:** Luca Palazzese, Debora Agata Anzalone, Federica Turri, Marco Faieta, Anna Donnadio, Flavia Pizzi, Paola Pittia, Kazutsugu Matsukawa, Pasqualino Loi

**Affiliations:** 1 Faculty of Veterinary Medicine, University of Teramo, Street R. Balzarini 1, Campus Coste Sant’Agostino, 64100 Teramo, Italy; 2 Institute of Agricultural Biology and Biotechnology (IBBA), National Research Council (CNR), 26900 Lodi, Italy; 3 Faculty of Bioscience and Technology for Food, Agriculture and Environment, University of Teramo, 64100 Teramo, Italy; 4 Department of Pharmaceutical Sciences, University of Perugia, 06123 Perugia, Italy; 5 Faculty of Agriculture and Marine Science, Kochi University, Kochi, 783-8502 Japan

**Keywords:** Biotechnology, Developmental biology

## Abstract

Freeze-dried spermatozoa typically shows a reduction in fertility primarily due to the DNA damage resulting from the sublimation process. In order to minimize the physical/mechanical damage resulting from lyophilization, here we focused on the freezing phase, comparing two cooling protocols: (i) rapid-freezing, where ram sperm sample is directly plunged into liquid nitrogen (LN-group), as currently done; (ii) slow-freezing, where the sample is progressively cooled to − 50 °C (SF-group). The spermatozoa dried in both conditions were analysed to assess residual water content by Thermal Gravimetric Analysis (TGA) and DNA integrity using Sperm Chromatin Structure Assay (SCSA). TGA revealed more than 90% of water subtraction in both groups. A minor DNA damage, Double-Strand Break (DSB) in particular, characterized by a lower degree of abnormal chromatin structure (Alpha-T), was detected in the SF-group, comparing to the LN-one. In accordance with the structural and DNA integrity data, spermatozoa from SF-group had the best embryonic development rates, comparing to LN-group: cleaved embryos [42/100 (42%) versus 19/75 (25.3%), *P* < 0.05, SL and LN respectively] and blastocyst formation [7/100 (7%) versus 2/75 (2.7%), *P* < 0.05, SF and LN respectively]. This data represents a significant technological advancement for the development of lyophilization as a valuable and cheaper alternative to deep-freezing in LN for ram semen.

## Introduction

Semen lyophilization is a promising technique that might be a cheap alternative to long-term storage in liquid nitrogen. The first significant result of this method was achieved by Wakayama and Yanagimachi^1^, that demonstrated for the first time the birth of healthy offspring from epididymal freeze-dried mouse spermatozoa. Since this pioneer work, dry spermatozoa successfully supported embryo development till the blastocyst stage in a wide range of experimental and farm animals: pig^2^, bovine^3^ and sheep^4^.

On the contrary of the first report in the mouse^1^, the number of offspring resulting from embryos produced with dry spermatozoa are scant and limited to only few species: rabbit^5^, rat^6^, horse^7^ and hamster^8^.

A justification for the higher developmental rates and offspring delivered in the original report^1^ might be found in the source of oocytes. While Wakayama and Yanagimachi^1^ used in vivo matured and ovulated mouse oocytes to test the developmental competence of freeze-dried mouse sperm, all the following studies involving freeze-dried sperm from other species invariantly used in vitro matured oocytes^2–4^.

However, the source of the oocytes and the culture methods alone cannot explain the difficulties in obtaining viable offspring at acceptable frequencies following fertilization with dry spermatozoa. A major problem is the occurrence of single and double strand DNA breaks as a consequence of the freeze-drying process. Our previous work has reported a clear negative correlation between the extent of DNA damage in dry spermatozoa and embryo cleavage, with developmental arrest with double strand breaks (DSBs) exceeding 2%^9^.

With the introduction of assisted reproductive technology (ART), some advanced techniques as flow cytometry (FCM) have been applied in several studies to provide accurate and unbiased evaluation of sperm functions, such as sperm DNA integrity^10–12^. Sperm DNA damage, as well as chromatin structure alterations extrapolated from the Sperm Chromatin Structure Assay (SCSA) are considered the most valuable parameters for the assessment of male fertility^10^. With SCSA, sperm samples are diluted and then acid-treated to melt the DNA strands at damaged sides. The DNA intercalating fluorescent dye acridine orange allows us to distinguish between single versus double stranded denatured DNA from native DNA regions.

Many factors influence the freeze-drying process, and it is not clear how it affects sub-cellular compartments of spermatozoa, the nuclear one in the first instance, and how it might differ between different species. Clearly, a sound understanding and control of the lyophilization process would result in an increased production of embryos and/or live offspring. It has to be recognized that the current lyophilization procedures follow closely the protocols used to dry foodstuff or pharmaceutical products. In particular, the standard approach adapted to spermatozoa is to plunge them directly into liquid nitrogen (LN)^13^ prior vacuum sublimation of the water fraction.

Dry storage of male gametes is in its infancy, therefore, the protocols originally used need to be inflected to the special needs of nucleated cells. Lyophilization threatens DNA stability through the physical damage exerted by the rapid cooling to − 196 °C, followed by the vacuum extraction of water. With this in mind, in this study we started to challenge the current protocols by skipping the deep-freezing step. In freeze-drying, sample freezing inflicts severe mechanical/osmotic stress, since no canonical cryoprotector, such as glycerol and ethylene glycol, can be added before freezing, as they are liquid at room temperature, therefore not suitable for dry storage. Here we have instead resorted to a controlled cooling (1 °C/min), until − 50 °C, prior to start the sublimation phase, and assessed the outcomes of the process in terms of spermatozoa’s residual water, structural and DNA stability and in vitro developmental of sheep oocytes injected with re-hydrated spermatozoa. Finally, we also monitored the fertility of dried spermatozoa after long-term storage.

## Results

### Residual moisture content and system stability in freeze-dried spermatozoa

**Figure 1:**
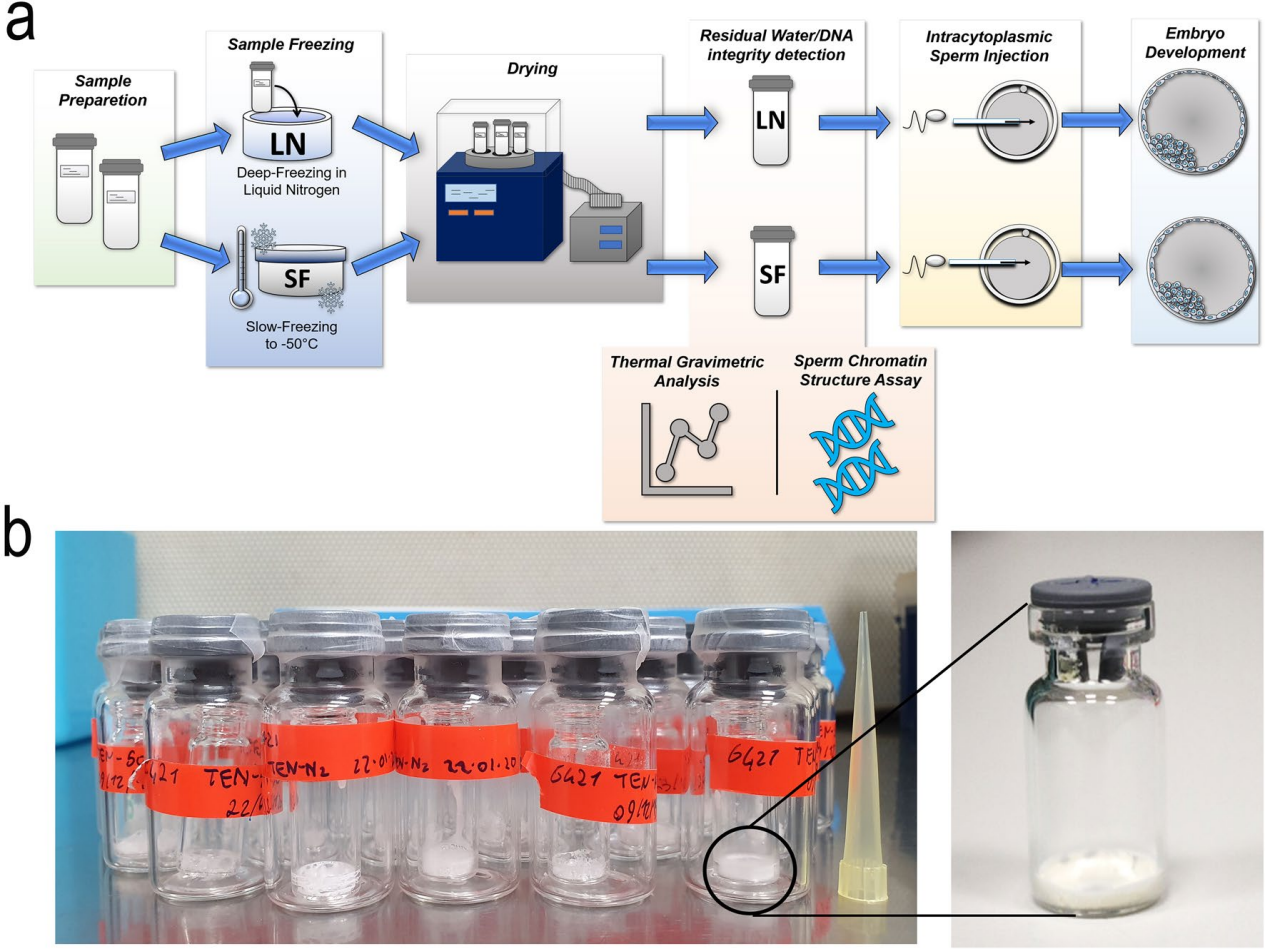
Experimental design. (**a**) The semen was frozen in two different methods before the lyophilization: Rapid-freezing, where the semen is plunged directly into liquid nitrogen (LN-group); Slow-freezing, where the sample is progressively cooled to a final temperature of − 50 °C with a cooling rate of 1 °C/min (SF-group). The influence of the two lyophilization methods on the spermatozoa quality were assessed by Thermal Gravimetric Analysis (TGA) to quantify the residual water and Sperm Chromatin Structure Assay (SCSA) to evaluate the DNA fragmentation. Fertilizing capacity of the both LN/SF—groups was tested by Intracytoplasmic Sperm Injection (ICSI) and evaluated the embryonic development. (**b**) On the left, freeze-dried spermatozoa contents in one-in-one glass vacuum sealed (glass vial of Ø 8 mm in glass vial of Ø 20 mm). On the right, freeze-dried spermatozoa in glass vial of Ø 8 mm.

The residual water detected in freeze-dried spermatozoa (Fig. 1) was determined by thermal gravimetric analysis (TGA). Data not differ significantly between LN and SF—groups and averaged 10.5% for both groups.

### Effects of the freeze-dried protocol on DNA integrity in lyophilized spermatozoa

The type of DNA damage was quantified with the following parameters: (a) mean alpha-T; quantifies the degree of abnormal chromatin structure with an increased susceptibility to acid-induced denaturation (indicated by the shift from green—stable, dsDNA—to red—denatured, ssDNA fluorescence) and is expressed as the ratio of red to total fluorescence intensity [red/(red + green)]. (b) Standard Deviation of Alpha-T both in Single-Strand Breaks (alpha-T_SD_SSBs) than Double-Strands Breaks populations (alpha-T_SD_DSBs), that shows the extent of abnormality in chromatin structure within a population. (c) Single-Strand Breaks Count (SSBs %), represents the percentage of Single-Strand Breaks on the total number of sperm cell acquired. (d) Double-Strand Breaks Count (DSBs %) represents the percentage of DSB on the total number of sperm cell acquired.

The sperm chromatin structure, assessed by Sperm Chromatin Structure Assay (SCSA), seems to not be affected by the freeze-dry protocol (Table 1). However, a minor trend was found in the degree of abnormal chromatin structure in Double-Strand Breaks population (mean alpha-T_DSBs) in SF-group.

**Table 1:** Sperm Chromatin Structure Assay (SCSA) on freeze-dried spermatozoa of LN and SF groups (LSM ± SEM).

Flow-cytometric variables	Freeze-dry protocols (FP)		FP effect	Ram effect
	LN	SF		
Alpha-T_DSB	0.4096 ± 0.0033	0.4091 ± 0.0033	ns	ns
ATSD_DSB	0.0135 ± 0.0007	0.0135 ± 0.0007	ns	*
Alpha-T_SSB	0.4661 ± 0.0036	0.4701 ± 0.0036	ns	ns
ATSD_SSB	0.0178 ± 0.0020	0.0212 ± 0.0020	ns	ns
SSB (%)	5.88 ± 0.69	4.42 ± 0.69	ns	ns
DSB (%)	86.36 ± 0.79	89.42 ± 0.79	ns	***

Mean alpha-T_DSBs = is indicative of the shift from green (stable, dsDNA) to red (denatured, ssDNA) fluorescence and is expressed as the ratio of red to total fluorescence intensity [red/(red + green)] of the Double-Strand Breaks; alpha-T SD_DSBs = alpha-T standard deviation of the Double-Strand Breaks; mean alpha-T_SSBs = is indicative of the shift from green (stable, dsDNA) to red (denatured, ssDNA) fluorescence and is expressed as the ratio of red to total fluorescence intensity [red/(red + green)] of the Single-Strand Breaks; *alpha-T_SD_SSBs* alpha-T standard deviation of the Single-Strand Breaks, *SSBs (%)* Single-Strand Breaks Count, *DSBs (%)* Double-Strand Breaks Count.

*Means probability of significant effect because of freeze-dry protocol and ram (*P < 0.05; ***P < 0.001).

**Figure 2:**
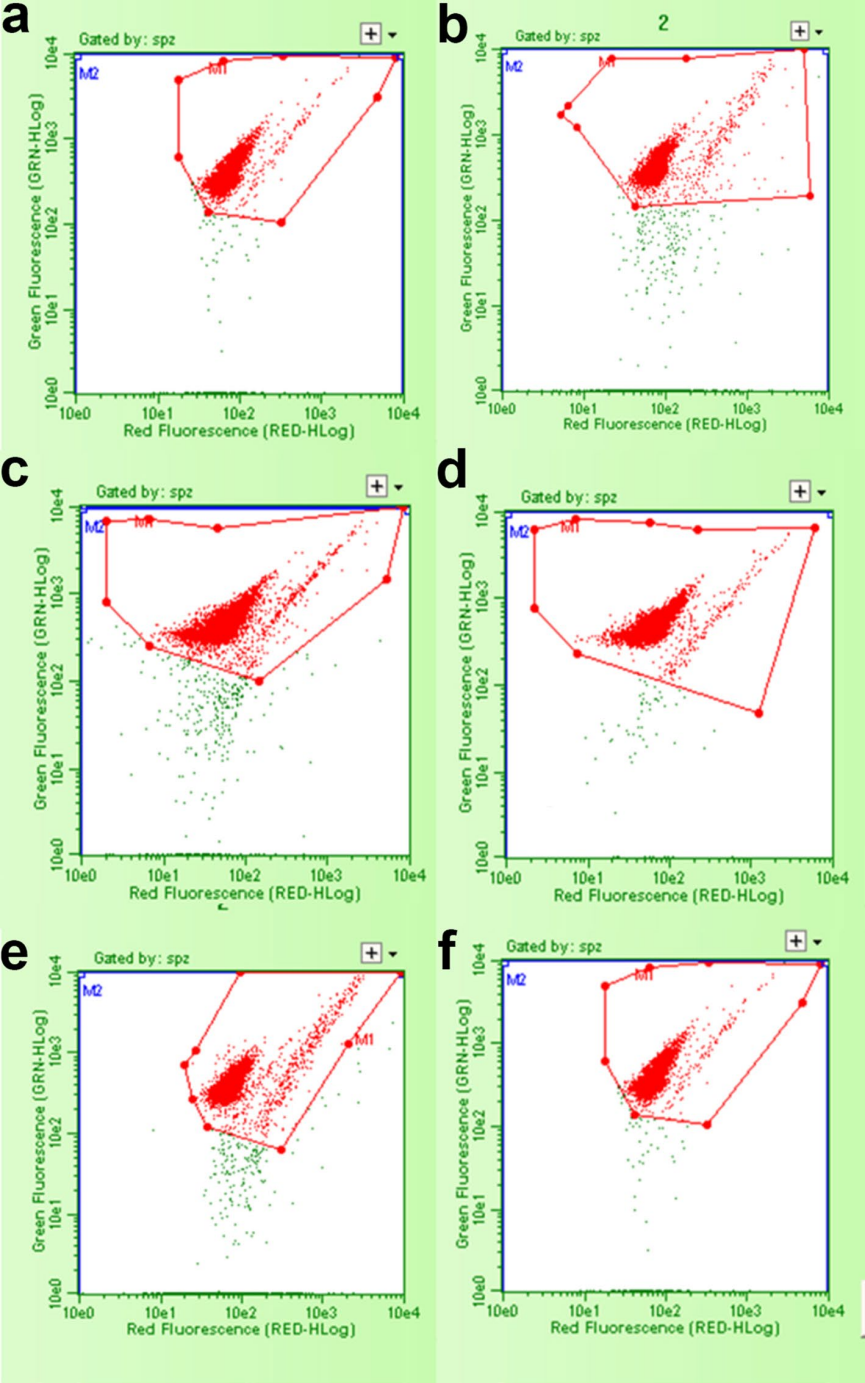
Representative green *vs* red fluorescence intensity scatter plots, obtained by flow cytometric analysis with SCSA of freeze-dried spermatozoa for each ram, in LN and SF—groups. The red dots in the octagons represent the Double Strand Break population (DSBs %) on the left, and the Single Strand Break population (SSBs%) on the right. The green dots outside the octagons are not sperm events and are considered as debris. (**a**) DSBs (%) and SSBs (%) distribution of Ram #1, LN-group; (**b**) DSBs (%) and SSBs (%) distribution of Ram #1, SF-group; (**c**) DSBs (%) and SSBs (%) distribution of Ram #2, LN-group.; (**d**) DSBs (%) and SSBs (%) distribution of Ram #2, SF-group; (**e**) DSBs (%) and SSBs (%) distribution of Ram #3, LN-group; (**f**) DSBs (%) and SSBs (%) distribution of Ram #3, SF-group.

The analysis of variance revealed that rams were a significant source of variation in DSBs (%) related to the freeze-dry protocol (Fig. 2). The standard deviation in DSBs (alpha-T SD_DSBs) and the percentage of DSBs were higher in SF than LN-group taking into account the individual ram effect (Table 1). In particular Ram #2 and Ram #3 had a considerably significant higher percentage of DSBs compare to Ram #1 (Ram #2, 93.57 ± 0.97; Ram #3, 93.97 ± 0.97 *vs* Ram #1, 76.13 ± 0.97;) and consequently significant lower percentage of SSBs (Ram #2, 5.80 ± 0.85; Ram #3, 2.04 ± 0.85 *vs* Ram #1, 7.61 ± 0.85).

### Slow-freezing preserve sperm morphology and acrosome caps after rehydration

**Figure 3:**
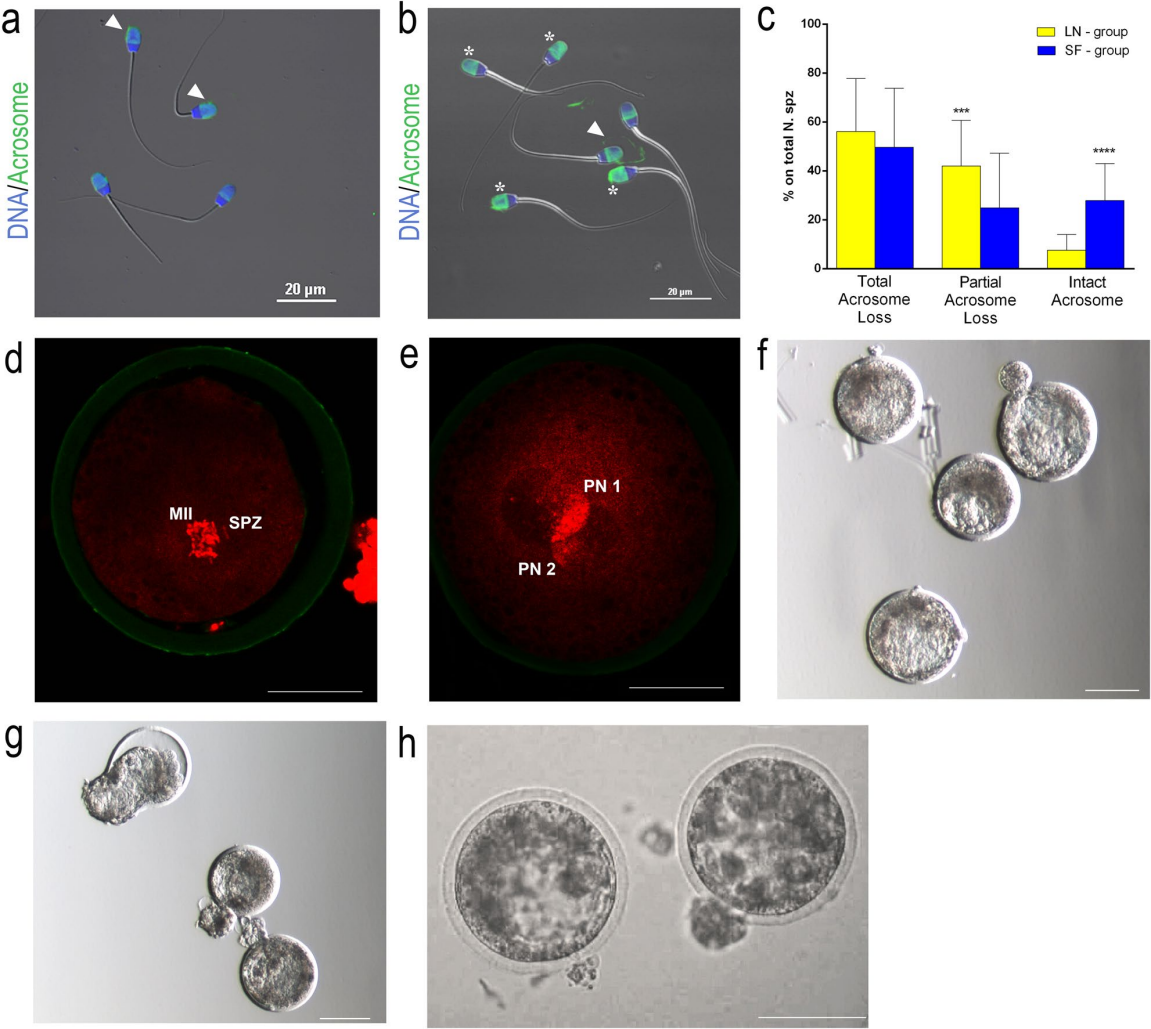
Acrosome integrity and embryonic development outcome. (**a**–**c**) Acrosome integrity by PSA, (**a**,**b**) blue DNA and green acrosome. (**a**) Spermatozoa of LN-group. (**b**) Spermatozoa of SF-group. In (**a**,**b**): asterisk marks intact acrosome; arrowhead marks partial acrosome loss, no labelling for total acrosome loss. (**c**) PSA outcomes: in LN-group n = 178, in SF—group n = 346; ***means *P* = 0.0002, ****means *P* < 0.0001. (**d**,**e**) Pronuclear formation after ICSI. The formation of two pronuclei (2PN) has been analyzed around 14–16 h post oocyte activation. (**d**) Non-activated oocytes, metaphase II plate (MII) and a non-decondensed sperm head (SPZ). (**e**) Activated oocytes with two clearly distinguishable pronuclei: Pronuclear 1 (PN 1) and Pronuclear 2 (PN 2). DNA is in red and zona pellucida in green. (**f**–**h**) Embryonic development following ICSI with freeze-dried spermatozoa. (**f**) Blastocyst produced by LN spermatozoa. (**g**) Blastocyst derived from SF-group stored for 3 months. (**h**) Blastocysts produced by freeze-dried spermatozoa stored for 1.5 year. Scale bar in (**a**,**b**) represents: 20 µm. Scale bar in (**d**,**e**) represents: 50 µm. Scale bar in (**f**–**h**) represents 100 µm.

The Pisum sativum agglutinin (PSA) test showed a higher proportion of spermatozoa with acrosomal partial loss in LN-group (42.7% vs 26.3%, LN and SF respectively, *P* = 0.0002); while full acrosome was detected in higher frequencies in the SF-group (9.6% vs 24.9%, LN and SF respectively, *P* < 0.0001) (Fig. 3a–c).

### Embryonic development following ICSI higher in SF-group

Our first care was to exclude that chemical activation would lead to the generation of haploid partonogenetic embryos. As reported in Table 2, the number of 2-pronuclear stage zygotes (2PN) did not statistically differ between frozen/thawed, LN and SF group. A representative figure of a 2PN zygote is reported in Fig. 3d,e. Oocyte fertilization by ICSI was used as a functional assay to test the developmental potential of re-hydrated spermatozoa of both groups. As anticipated by DSB percentage, the oocytes receiving a SF-spermatozoa—stored for 1–3 months—started to cleave in higher proportion comparing to LN ones (cleavage 42% vs 25.3%, SF and LN respectively, *P* < 0.05) (Table 3). Likewise, blastocyst development by day 7 after ICSI was higher in the SF (Table 2, Fig. 3f,g) (7% vs 2.7% on total number of injected oocytes in SF and LN respectively, *P* < 0.05). On the other side, the blastocyst rate was higher in untreated control (frozen/thawed semen) than LN-group (14.4% vs 2.7%, frozen/thawed and LN respectively, *P* < 0.05) although without a significant difference respect to the SF-group (Table 3).

**Table 2:** Pronuclear formation after ICSI with frozen/thawed and freeze-dried spermatozoa evaluated at 15–16 h post activation.

Groups	2PN (%)
Frozen/thawed	21/26 (80.7)
LN-group	20/26 (76.9)
SF-group	21/27 (77.7)

2PN = 2 Pronuclei stage.

**Table 3:** Embryo development of sheep oocytes injected with frozen/thawed and freeze-dried spermatozoa treated in LN and in slow-freezing (SF) stored 1–3 months and stored for 1 months and 1.5 year.

Treatment and storage	N. Oocytes	Cleavage (%)	Expanded blastocysts (%)
Frozen/thawed	76	30 (39.5)	14 (14.4)^b^
LN-group	75	19 (25.3)^a^	2 (2.7)^b,c^
SF-group	100	42 (42)^a^	7 (7)^c^
SF-group—1 months	132	47 (35.6)^d^	10 (7.6)^e^
SF-group—1.5 year	101	16 (15.8)^d^	2 (2)^e^

The cleavage (only the 2-cell stage embryos were considered as cleaved) and the blastocyst formation was evaluated at 24 h and 7 day after activation, respectively.

Values with similar superscript differs significantly (*P* < 0.05).

### Positive association between DNA integrity parameters and embryo development variables on freeze-dried spermatozoa

A significant and positive correlation between some DNA integrity parameters and embryo development was found. Mean alpha-T_DSBs showed a strong negative correlation with its respective alpha-T SD_DSBs (r =  − 0.90, *P* = 0.0149). On the contrary, the degree of abnormal chromatin structure in Single-Strand Breaks population (mean alpha-T_SSBs) showed a strong positive correlation with its respective standard deviation (alpha-T SD_SSBs) (r = 0.92, *P* = 0.0077).

The percentage of SSBs displayed a strong negative relationship with the percentage of cleavage and blastocyst developmental rates (r = 1.00, *P* < 0.0001). Cleavage and blastocyst percentage combination (%) showed a strong linear relationship (r = 1.00, *P* < 0.0001).

### Decline of developmental potential of freeze-dried ram spermatozoa after long-term storage

Given the better findings obtained in SF spermatozoa, we elected them for long-term storage in vacuum-sealed glass vials for 18 months. SF spermatozoa re-hydrated after 18 months (long-term storage) and 3 months (control) were injected into metaphase II oocytes, and the development following artificial activation were monitored at two time points: first cleavage and blastocysts stage. Both time points check revealed a developmental decline for the log-term stored semen: cleavage (15.8% vs 35.6%, *P* = 0.0010, long-term storage vs control respectively), and blastocyst development (2% vs 7.6%, *P* = 0.0097, long-term storage vs control respectively) (Table 3, Fig. 3h).

## Discussion

Here we have demonstrated for the first time that a mild sub-zero temperature prior water subtraction better preserves structure, DNA integrity and in turn developmental potential of freeze-dried ram spermatozoa. Besides the demonstration that SF lyophilization offer advantages over deep cooling lyophilization, we have also expanded our knowledge on the long-term storage of dry ram spermatozoa.

As already stated, in vitro and in vivo development of embryo produced by ICSI with dry spermatozoa is unsatisfactory for species other than the mouse. Previous reports^3,14^ including from our group^9^, have identified in the DNA damage the major hampering factors. Even though the oocyte has a remarkable DNA repairing capacity^15^, it is likely that point mutations are incorporated genome-wide as a result of DNA repair activities, thus undermining the further development. Therefore, every technicality capable to reduce DNA damage during the freeze-drying process should automatically result in improved fertilization outcomes. Spermatozoa lyophilization is effectively in its ground state therefore, there is ample room for improvement, as our finding demonstrate.

Another important factor is the residual moisture of the dry sample, as it can provide an idea on the efficiency of the freeze-drying process (quantity of water removed during sublimation) and confer stability to the dry state. To this extent, it is crucial to leave in the spermatozoa the fraction of “bound water”, or “structural water” that binds covalently to proteins, lipids and DNA^16^, otherwise, a structural shrinkage could occur, with massive damaging effects, especially on DNA. Furthermore, Jiang and Nail^17^ reported a critical decrease of the recovery activity after freeze-drying for different proteins when residual moisture values were lower than 10%. We do not know the relative amount of bound water in freeze-dried spermatozoa, although some indications might be provided by anhydrobiotic organisms, like midge or tardigrades, where bound water account for 2–3% of the total content^18,19^. Hence, a fine calibration of the residual water is crucial for successful and safe drying. The quantification of residual humidity in lyophilized semen has not been estimated with precise analytical methods so far, even though the issue started to come under the spotlight^20,21^. In this work we evaluated the moisture residual in our freeze-dried samples using Thermal Gravimetric Analysis (TGA), one of the most sensitive assays for residual water detection in lyophilized samples. The data showed a residual water slightly higher than 10% in both LN and SF-samples. In bull spermatozoa the residual water estimation was carried out through gravimetrical analysis^22^ and their data report a percentage of residual water of about 3%. The explanation for a higher reduction in the free water of Hara's and collaborative work lead in a different temperature program in the primary and secondary drying steps. Moreover, Hara et al.^22^ reduced the concentration of EGTA in favour of trehalose, causing an increase of the system Tg’ (glass transition temperature of the maximally freeze-concentrated phase) and hindering the collapse of the matrix assuring an efficient sublimation in the primary drying step. On the other hand, the effect of calcium chelating agents, outweighs as endonuclease activity inhibitors in freeze-dried spermatozoa preserving DNA integrity^23^, justifying their use in the freeze-drying method. The same approach has been adopted in this work.

We knew from previous reports that freeze-dried ram spermatozoa preserve a good morphology^4^. In this work we used the acrosomal membrane as a marker of structural integrity. Only a small proportion of LN spermatozoa showed acrosome integrity (9.6%), on the contrary of SF ones, where intact acrosomes were detected in 26.3% of the spermatozoa (Fig. 2a–c). Acrosome loss in LN spermatozoa clearly arises from the sudden intracellular ice crystals formation on larger surface area, a typical finding in rapid-freezing, that lead to membrane structural instability^24^.

It is established that standard lyophilization heavily damages DNA, compromising the spermatozoa developmental potential^3,14^. In epididymal freeze-dried ram spermatozoa, as little as 2% of DSB widely distributed completely arrest embryo development^9^. Therefore, ultra-sensitive assays should be used to investigate DNA damage in dry spermatozoa. Generally, DNA status in lyophilized semen was performed using Sperm Chromatin Dispersion (SCD), Single cell gel electrophoresis (SCGE) or comet assay and SCSA^9,25,26^. However, the DNA parameters derived from SCD and SCGE analysis exhibited ample variation in each replicate, making an infeasible correlation with the fertilizing capacity of dry spermatozoa^25^. In our study, the percentage of SSB determined by SCSA shown a strong correlation with the fertilizing capacity of the dry spermatozoa, both in term of cleavage and blastocyst production. Our DNA damage data agrees with published work on lyophilized horse spermatozoa^27^.

The in vitro functional assays, the fertilization by ICSI of in vitro matured sheep oocytes with SF and LN re-hydrated spermatozoa, indicated a neat supremacy of the first ones in directing embryonic development, thus confirming the structural and DNA integrity data (Table 3). The blastocyst rate obtained, 7%, is in line with data gathered in other farm animals^7,28,29^.

Finally, we tested for the first time the fertilization capability of freeze-dried ram spermatozoa after long term storage. Table 3 unquestionably underlines a strong decrease in spermatozoa fertility, with 8.8% blastocyst with “fresh” spermatozoa (1–3 months of storage), up to 2% after long-term storage (up to 18 months). The data clearly indicate a remarkable reduction in the spermatozoa fertilizing capability owing very likely to unsuitable storage conditions. So far, the best condition for long-term conservation of the freeze-dried spermatozoa still remains at − 80 °C^30^, which is still far away from room temperature storage, and associated with elevated costs. Probably, the need of cold storage at − 80 °C of dry spermatozoa is due to an excess of free water, as well as inadequate packaging. Normally, our samples are sealed in glass vials under vacuum, as shown in Fig. 1b; a saturation with inert gases, like Argon 90% /Helium 10%, inside the glass/stainless steel, as currently done for isolated nuclei acids (RNA and DNA), might ensure a better sample stability^31^.

To conclude, our work has demonstrated that a milder-temperature freezing of the spermatozoon prior drying preserves structural and DNA stability over the current procedure, and results in a significant improvement of their fertilizing potential. Furthermore, we have highlighted the fertility decay of spermatozoa following a relatively long storage under vacuum, indicating the need to work our suitable storing and packaging condition for dry spermatozoa. Overall, our contribution is a leap forward for the establishment of alternative, low cost biobanking solution for genetic banks for human infertility, animal breeding and biodiversity preservation.

## Material and methods

### Animals

All animal experiments (semen collection) have been approved by the Italian Ministry of Health, upon the presentation of the research description prepared by the ethics committee of the Istituto Zooprofilattico Sperimentale di Teramo (Prot. 944F0.1 del 04/11/2016). The number of the authorization granted by the Italian Ministry of Health is no 200/2017-PR. All methods were performed in accordance with the relevant guidelines and regulations of the Italian Minister of Health.

### Chemicals

Unless otherwise stated, all materials used were purchased from Sigma Aldrich (St Louis, MO, USA).

### Semen collection

Semen was collected from three adult fertile Sardinian rams using an artificial vagina filled with warm water (40–44 °C) and connected to a 15 ml sterile tube (Corning). Immediately after collection, sperm motility was evaluated using a stereomicroscope and transported to the laboratory using a transportable incubator (INC-RB1 Biotherm, Cryologic, Blackburn, Australia) at 32–35 °C.

### Semen cryopreservation

As untreated control we used frozen/thawed ram semen. The spermatozoa cryopreservation was performed as previously reported^32^. Briefly, Medium A [67.20% of Basic Medium (300 mM TRIS base, 105 mM citric acid, 82 mM fructose, 150,000 IU penicillin G, 2 mM streptomycin in (67.20 ml) double-distilled water (ddH20)), 20% of egg yolk, 12.8% of ddH2O] and Medium B (67.20% of Basic Medium, 20% of egg, yolk, 12.80% of glycerol) were added in equal volumes to the ejaculate to reach a final concentration of 400 × 106 spermatozoa/ml. Medium A was added to the semen first and transferred to 4 °C for 2 h. Subsequently, Medium B was then added to the suspension and left for further 2 h at 4 °C. Next, 250 μl straws were filled, sealed with polyvinyl alcohol (PVA), placed onto a metallic grid, and stabilized at 4 °C for 2 h and finally immersed in liquid nitrogen.

### Sperm lyophilization

To select the mobile fraction, the extended semen underwent swim up. Briefly, 20 µl of ejaculate were placed to the bottom of a 15 ml sterile tube containing 1.5 ml of Basic Medium (TRIS 3.6% w/v, Citric Acid 2% w/v, Fructose 1.5% w/v, Penicillin G 100.000 IU, Streptomycin 0.1% w/v) and left inclined at 45° for 20 min at 37 °C. Then, 750 µl of the supernatant containing swim-upped spermatozoa, was harvested and the concentration was corrected to reach 20 × 10^6^ spermatozoa/ml in the final lyophilization medium [1 ml 0.5 M TRIS (in water), 5 ml 0.5 M EGTA (in water), 2.5 ml 1 M NaCl (in water)]. After this, the semen was processed for freeze-drying according to the following methods (Fig. 1a): (i) Spermatozoa were lyophilized as previously reported^4^. Briefly, spermatozoa diluted in lyophilization medium, were aliquoted in 100 µl each vial and plunged directly into liquid nitrogen for 2–3 min until solution was visibly frozen (LN-group). (ii) 100 µl of diluted semen was aliquoted into glass vials (Ø 8 mm), then loaded into Mister Frosty (Thermo Fisher Scientific) to be progressively cooled to a final temperature of − 50 °C (SF-group) with a cooling rate of − 1 °C/min.

At the end of freezing step, the glass vials containing frozen samples of both groups were inserted into a wider glass vial (Ø 20 mm) and placed inside the Freeze-Dry apparatus (SP Scientific-VirTis, 2.0 BenchTop) with the condenser at a temperature of − 58 °C and the freeze-drying chamber at − 12 °C. As soon as the vials are placed in the lyophilizer, the vacuum pump starts and made to work for 16 h until when each ampule was sealed under vacuum condition (pressure 15 µBar) (Fig. 1b) and the temperature of the freeze-drying chamber reaches − 30 °C. The samples produced were stored for a different time: a portion (n = 30) of vials were stored for 1–3 months, and (n = 6) a part for 18 months, both at 4 °C in the dark.

### Residual water content measurement by thermal gravimetric analysis (TGA)

Thermogravimetric determinations were carried out in an airflow of 50 mL/min by a NETZSCH STA 449 Jupiter thermal analyzer connected to a NETZSCH TASC 414/3 A controller (NETZSCH Group, Selb, Germany). Analysis was carried out in the temperature range 25–160 °C at a heating rate of 10 °C/min.

### Sperm DNA fragmentation

As previously described in other our studies^32,33^, Sperm Chromatin Structure Assay (SCSA) was assessed using a Guava easyCyte 5HT microcapillary flow cytometer. The fluorescent probes were excited by a 20 mW argon ion laser (488 nm). Forward Scatter *vs* Side Scatter plots were used to separate sperm cells from debris. Non-sperm events were excluded from the analysis. Fluorescence detection was set with three photomultiplier tubes: detector FL-1 (green: 525/30 nm), detector FL-2 (yellow/orange: 583/26 nm), and detector FL-3 (red: 655/50 nm). Calibration was carried out using standard beads (Guava Easy Check Kit; Merck Millipore). A total of 5000 sperm events per sample for each ram were analysed at a flow rate 200 cells/s.

The SCSA test was performed using the sperm chromatin structure assay^10^ as previously described^32,33^ with some modifications, by using acridine orange, a fluorochrome that turns from red to green depending on the degree of chromatin compaction, to distinguish between denatured, single-stranded and double-stranded DNA regions. The vials with freeze-dried spermatozoa of each rams were rehydrated as described in Anzalone et al.^4^ by adding 100 μl of sterile bi-distilled water up and correct the semen concentration to 20 × 10^6^ spermatozoa/ml. 25 µl of the re-hydrated freeze-dried sperm cells suspension were diluted in 195 μl of TNE buffer (0.01 M Tris–HCl, 0.15 M NaCl, 1 mM ethylenediaminetetraacetic acid (EDTA), pH 7.4) and added to 1200 μl of an acidic solution (Triton X-100 0.1%, 0.15 M NaCl, 0.08 M HCl; pH 1.2). After 30 s, cells were stained with 1.2 ml of acridine orange solution (6 μg/ml in 0.1 M citric acid, 0.2 M Na2HPO4, 1 mM EDTA, 0.15 M NaCl; pH 6). After 2.5 min, two replicates per sample were read on the flow cytometer.

Data were acquired and analysed using respectively cytoSoft and EasyCompDNA software (Merck KGaA, Darmstadt, Germany; distributed by IMV Technologies) and expressed as: mean alpha-T of the Single (mean alpha-T_SSBs) and Double Strand Breaks (mean alpha-T_DSBs); Standard Deviation of alpha-t of the Single (alpha-T_SD_SSBs) and Double Strand Breaks (alpha-T SD_DSBs; Single-Strand Breaks Count (SSBs%) and Double-Strand Breaks Count (DSBs %).

### Acrosomal integrity: *Pisum sativum* agglutinin (PSA)

Freeze-dried spermatozoa from LN and SF groups were rehydrated with 100 µl of bi-distilled water and washed in PBS for 5 min at 3000 rpm at room temperature (RT). After the washing, a smear was made with 10 µl of semen suspension. The slides were dried at room temperature for 5 min and fixed in 100% ethanol for 5 min. The slides where then washed 2–3 times in clean PBS and stained with 200 µl of 1:1 PSA:DAPI (PSA 40 mg/ml, DAPI 0.5 µg/ml, both in PBS) 12 min, RT, in the dark. The slides were fast-washed 2–3 times in clean bi-distilled water and mounted with Fluoromount aqueous mounting medium and analyzed under confocal microscope (Nikon Eclipse Ti-E, Software: NIS-Elements Confocal software). The acrosome integrity was scored in three different levels: total acrosome loss, with the total absence of the acrosome; partial acrosome loss, with partial removal of the acrosome; intact acrosome, totally intact acrosome (Fig. 3a,b).

### Oocyte recovery and in vitro maturation (IVM)

Sheep ovaries were obtained from a local slaughterhouse and transferred at 37 °C to the laboratory within 1–2 h from slaughter. Cumulus-Oocyte Complexes (COCs) were aspirated using a 21 G needles in the presence of 4-(2-hydroxyethyl)-1-piperazineethanesulfonic acid (HEPES) buffered TCM-199 medium (Gibco, Life Technologies, Milan, Italy) and 0.005% (w:v) heparin. Only COCs having at least 2 or 3 layers of compact cumulus cells were selected for IVM, which was performed in 4-well dishes containing 500 μl of IVM medium per well. The IVM medium was composed of bicarbonate-buffered TCM-199 (Gibco) containing 2 mM glutamine, 0.3 mM sodium pyruvate, 100 μM cysteamine, 10% fetal bovine serum (FBS) (Gibco), 5 μg/ml follicle stimulating hormone (FSH; Ovagen, ICP, Auckland, New Zealand), 5 μg/ml luteinizing hormone (LH) and 1 μg/ml 17 β-estradiol. Maturation was completed in a humidified atmosphere at 38.5 °C and 5% CO2 in air for 24 h, as previously described^34^. After IVM, only MII oocytes with an expanded cumulus and normal morphology were selected for Intracytoplasmic Sperm Injection (ICSI). Cumulus was removed by fast pipetting of COCs into a 500 μl of 300 U/ml of hyaluronidase solution in Hepes-buffered TCM-199 + 0.4% BSA (w:v) (H199). Then, oocytes were 3 times washed in H199 and incubated into a Petri dish, until injection.

### Intracytoplasmic sperm injection (ICSI)

Only oocytes with visible I polar body (PB) were processed by ICSI. Freeze-dried spermatozoa of LN and SF groups were rehydrated by adding 100 μl of sterile bi-distilled water. Then, a 5 μl aliquot was suspended in 100 μl of H-199 + 0.4% BSA (w:v). The latter was diluted 1:1 with 12% (w:v) polyvinylpyrrolidone (PVP) in PBS. Finally, 3 drops of 10 μl drops were placed on the lid of a Petri dish on a warmed microscope stage and covered with mineral oil. ICSI was performed according to Anzalone et al.^35^ with slight modification. Briefly, ICSI with frozen/thawed and freeze-dried spermatozoa was carried out on an inverted microscope (Nikon Eclipse E-800) connected to a micromanipulation system (Narishige NT-88NEN, Tokyo, Japan), using a piezo-driven micropipette system (PiezoXpert, Eppendorf, Milan, Italy). At 24 h from the start of IVM, the matured oocytes were injected. PVP/sperm-containing drops were renewed every ten injected oocytes. After injection, the oocytes were chemically activated by 5 min incubation in 5 μM ionomycin dissolved in H199 + 0.4% BSA and placed in embryo culture as described in the following paragraph.

### Pronuclear staining

Pronuclear visualization was performed as previously reported^4^. Briefly, a total number of 26, 26 and 27 presumptive zygotes of frozen/thawed, LN and SF- group respectively, were fixed in 4% paraformaldehyde (PFA) for 20 min at RT, 14–16 h post oocyte activation. Then, presumptive zygotes were permeabilized with 0.1% Triton X-100, washed twice in 0.4% PVP in PBS and stained with 40 μg/ml PSA-FITC and 5 μg/ml Propidium Iodide, 8 min in the dark, RT. Thus, presumptive zygotes were washed twice in 0.4% PVP in PBS, mounted on the slides and observed under the confocal microscope (Nikon Eclipse Ti-E, Software: NIS-Elements Confocal software).

### Embryo culture

The embryo culture was performed following our lab’s state^4^. Briefly, all presumptive zygotes that resulted from oocytes fertilized by ICSI, were cultured in numbers of 4 or 5 per drop in 20 μl of SOF-enriched with 2% (v:v) basal medium Eagle essential amino acids (EAA), 1% (v:v) minimum essential medium (MEM) non-essential amino acids (NEAA) (Gibco), 1 mM glutamine, and 8 mg/ml fatty acid-free BSA, covered by mineral oil. The medium was renewed on day 3 of culture [SOF-supplemented with 0.27 mg/ml glucose (SOF+), 2% EAA, 1% NEAA]; again on day 5 of culture [SOF+ with 10% of charcoal stripped FBS (cs-FBS), 2% EAA, 1% NEAA]; and again on day 6 (1:1 MEM/M199 enriched with 10% cs-FBS, 2.5 μg/ml gentamicin and 1% sodium pyruvate) until day 7th or 8th of culture. The in vitro development was evaluated at 24 h post-activation for cleavage (only the 2 cell-stage embryos was considered to have cleaved) and at day 7th/8th of culture for expanded blastocyst formation. Embryos observation and images were acquired by Nikon Eclipse Ti2-U inverted microscope using Octax EyeWare Imaging Software (version 2.3.0.372).

### Statistical analysis

Acrosome integrity and embryo development data were handled in GraphPad Prism for Windows (Version 6.01, GraphPad software, CA, USA) using Fisher’s exact test and One-way Anova non-parametric test (for acrosome integrity and embryo development respectively). Tests were based on more than five replicates per experiment. Data are reported as means ± standard error mean (SEM).

TGA analysis were run in triplicate and the results reported as average.

Data obtained from SCSA assessment were analysed using the SAS package v 9.4 (SAS Institute Inc., Cary, NC, USA). The General Linear Model procedure (PROC GLM) was used to evaluate the effect of the freeze-dry protocols on the SCSA sperm quality parameters. The model included the fixed effect of the ram. Results are given as adjusted least squares means ± standard error means (LSM ± SEM). A PROC CORR was applied to calculate Spearman’s correlation coefficients within freeze-dried sperm quality and embryo development variables.

For all the tests the level of significance was set at *P* < 0.05.
